# Physical Activity during the First COVID-19-Related Lockdown in Italy

**DOI:** 10.3390/ijerph18052511

**Published:** 2021-03-03

**Authors:** Eszter Füzéki, Jan Schröder, Nicolò Carraro, Laura Merlo, Rüdiger Reer, David A. Groneberg, Winfried Banzer

**Affiliations:** 1 Division of Preventive and Sports Medicine, Institute of Occupational, Social and Environmental Medicine, Goethe-University Frankfurt, Theodor-Stern-Kai 7, 60590 Frankfurt, Germany; groneberg@med.uni-frankfurt.de (D.A.G.); banzer@med.uni-frankfurt.de (W.B.); 2 Department of Sports Medicine, Faculty for Psychology and Human Movement Science, Institute for Human Movement Science, University of Hamburg, Turmweg 2, 20148 Hamburg, Germany; jan.schroeder@uni-hamburg.de (J.S.); ruediger.reer@uni-hamburg.de (R.R.); 3 Center for Sports Medicine, Department of Prevention, ULSS2 Marca Trevigiana, 31100 Treviso, Italy; nicolo.carraro@aulss2.veneto.it (N.C.); laura.merlo@aulss2.veneto.it (L.M.)

**Keywords:** walking, cycling, leisure time activity, corona, confinement

## Abstract

The spread of the COVID-19 virus was met by a strict lockdown in many countries around the world, with the closure of all physical activity (PA) facilities and limitations on moving around freely. The aim of the present online survey was to assess the effect of lockdown on physical activity in Italy. Physical activity was assessed using the European Health Interview Survey questionnaire. A total of 1500 datasets were analyzed. Differences between conditions were tested with a chi^2^-based (χ^2^) test for categorical variables, and with the Student’s *t*-test for paired data. A fixed effects binary logistic regression analysis was conducted to identify relevant predictor variables to explain the compliance with World Health Organisation (WHO) recommendations. We found a substantial decline in all physical activity measures. Mean differences in walking and cycling metabolic equivalent of task minutes per week (METmin/week), respectively, were 344.4 (95% confidence interval (95% CI): 306.6–382.2; *p <* 0.001) and 148.5 (95% CI: 123.6–173.5; *p* < 0.001). Time spent in leisure time decreased from 160.8 to 112.6 min/week (mean difference 48.2; 95% CI: 40.4–56.0; *p* < 0.001). Compliance with WHO recommendations decreased from 34.9% to 24.6% (chi^2^ (1, 3000) = 38.306, *p* < 0.001, V = 0.11). Logistic regression showed a reduced chance (OR 0.640, 95% CI: 0.484–0.845; *p* = 0.001) to comply with WHO PA recommendations under lockdown conditions. Measures to promote physical activity should be intensified to limit detrimental health effects.

## 1. Introduction

The highly infectious coronavirus disease 2019 (COVID-19), which is caused by a novel coronavirus, SARS-CoV-2, has evolved to be a worldwide pandemic. Italy was one of the European countries which was hardest hit by the pandemic in the spring of 2020 [1,2,3]. In the 3 months from 15 February to 15 May 2020, an average excess mortality of 29.5% was registered for the whole country, with a rate of 400% in the northern provinces, which were the most severely affected by the pandemic [3]. Since, at the time of the outbreak of the pandemic, neither an effective preventive vaccine nor specific pharmaceutical options were widely available, traditional public health measures such as hygiene rules, isolation, social distancing, and quarantine were identified as successful strategies to contain the spread of the virus [4,5].

In the spring of 2020, full or partial lockdown was implemented in more than 100 countries worldwide, with Italy as the first country in Europe to introduce such measures [6]. On 21 February 2020, the regional authorities announced lockdown measures in selected municipalities in Veneto [7], and later in Lombardy (Northern Italy). On 9 March 2020, lockdown measures came into force across the whole country [3,8]. Lockdown in Italy was hard: only essential businesses were allowed to operate [9], and access to sports facilities, gyms, and public areas for leisure time physical activities (such as gardens and parks) was restricted [10,11]. Training and sports competitions were permitted only for the athletes competing at the national and international levels. Individual physical activity and walking dogs in the proximity of one’s home was allowed, with slightly different rules for each region (i.e., in Veneto, not farther than 200 m from one’s residence). Active transport on foot or by bicycle was possible for essential workers and to run non-deferrable errands, such as grocery and pharmacy shopping [3,8].

During the second wave in the autumn and winter of 2020, an increasing number of countries reinstated lockdown measures [6]. Based on mathematical modeling, it cannot be ruled out that that prolonged or intermittent social distancing may also be necessary into 2022 [12].

Ongoing or intermittent shelter-in-place orders severely impact daily life and can potentially influence all health-related behaviors, including physical activity (PA). Indeed, objectively measured (via the Argus app Azumio Inc. Palo Alto, CA, USA) step counts showed considerable decline worldwide following the pandemic declaration (11 March 2020), with a decline in mean step count of 27.3% 30 days after the declaration [13]. The analysis of BetterPoints smartphone app data in the United Kingdom in 5395 users showed a differentially altered PA behavior [14]. While 63% of users became less active between baseline and the first week of the lockdown, 16% maintained their activity level, and 21% increased their PA [14]. In a large sample of the Spanish adult population, the PA level was reduced by 20% following the lockdown [15].

Regular PA is crucial for maintaining good physical and mental health [16]. Sustained reduction of PA poses significant health challenges and should be avoided [17]. The aim of the present study was to assess the potential changes in PA in Italy following the lockdown measures in the spring of 2020. We hypothesized that overall activity levels declined and the number of respondents who complied with current PA recommendations also reduced.

## 2. Materials and Methods

### 2.1. Study Design and Procedure

We conducted a cross-sectional online survey between 15 April and 23 June 2020 in the Italian general population. The survey was performed using the SoSci Survey tool (SoSci Survey GmbH, Munich, Germany). All participants provided informed consent. Prior to launching the survey, an ethical approval by Goethe University, Frankfurt was obtained (reference number 2020-18).

The link to the survey was distributed via the Local Health Authority of Treviso (Azienda ULSS 2 Marca Trevigiana) through the following channels—the Local Health Authority of Treviso (AULSS 2) posted an official press release containing the presentation of the study and the link to the survey on its website, and distributed it through its social media. The contents have been relaunched by local newspapers, websites, and personal social media. We also contacted the school authorities of the province of Treviso, via phone calls and email, and invited them to share the survey with the families of the students and to their staff.

### 2.2. Questionnaire

The questionnaire consisted of two major thematic parts: one on habitual PA and one on the use of, and attitude towards, virtual PA offers. The current article reports on habitual PA only. Parts covering self-perceived health (HS1–HS3), anthropometric data (BM1–2), and PA (PE1–PE8) were identical with the respective questions from the European Health Interview Survey (EHIS wave 2) [18]. Wording was identical with the official Italian translation. The EHIS PA questionnaire has shown acceptable-to-good reliability and validity [19]. Questions relating to PA were asked twice, once with reference to pre-lockdown (“normal”) conditions, and once with reference to lockdown (“lockdown”) conditions. Educational attainment was assessed according to the International Standard Classification of Education (ISCED 2011) [20] and occupation according to the European Skills, Competences, Qualifications and Occupations classification (ESCO) [21].

### 2.3. Data Processing and Statistical Analysis

PA data were scored according to the official EHIS scoring protocol [22]. In short, the following PA outcomes were calculated: (a) work-related PA (WRPA), (b) transport-related PA (transport-related walking and cycling minutes per week; TRPA), (c) leisure time PA (total minutes of sports, fitness, and recreational leisure time activities in at least 10 min bouts per week; LTPA), (d) days of muscle-strengthening activities per week (DMSA), and (e) compliance with World Health Organization (WHO) PA recommendations [23] (aerobic activities > 150 min/week determined from LTPA and cycling from TRPA, and ≥ two days’ muscle-strengthening activities/week; active vs. inactive).

Self-reported body weight and height were used to calculate body mass index (BMI).

Data are presented as frequencies (categorical variables), and as mean and standard deviation (SD) (scaled parameters).

Differences between conditions were tested with a chi^2^-based (χ^2^) test for categorical variables with Cramer’s V as a measure for the effect size in the case of significant differences. Small, moderate, or large effect sizes were indicated by V = 0.1, V = 0.3, and V = 0.5, respectively. For scaled datasets, mean differences between conditions were analyzed using the Student’s t*-test for paired data, including the respective confidence interval for difference of *means (95% CI) and effect size (Cohen’s D). Small, moderate, or large effect sizes were indicated by |d| = 0.2, |d| = 0.5, and |d| = 0.8, respectively. Frequencies (counts and percent values) were described for changes in PA subdivided into increases, no changes, or decreases from the normal to the lockdown condition separately for participants complying and not complying with WHO PA recommendations prior to the lockdown.

In order to identify relevant predictor variables to explain the compliance with WHO recommendations (0 = inactive/1 = active), a fixed effects binary logistic regression analysis was conducted using the following measures: work related physical activity (WRPA) (work effort: 0 = no task (new encoded), 1 = sitting/standing, 2 = moderate, and 3 = demanding interpreted as scaled variable), transport related physical activity (TRPA), leisure time physical activity (LTPA), and day of muscle strengthening activities (DMSA) interpreted as scaled variable, and demographic data including sex (0 = male, 1 = female), age, and BMI. Additionally, the “normal” and “lockdown” condition was entered as a categorical variable (encoded as 0 = non-normal = lockdown, 1 = normal).

All statistical analyses were computed using IBM SPSS software, V22.0 (IBM, Armonk, NY, USA). Significance was accepted for *p*-values ≤ 0.05.

## 3. Results

### 3.1. Descriptive Statistics

After removing five datasets because of an age below 15 years, 1500 datasets (n = 1126; 75.1% females and n = 368; 24.5% males) were included into the detailed analysis. Participants were 43.1 ± 11.3 years old and had a BMI of 23.7 ± 4.0. Of the total number of respondents, 4.7%, 64.1%, 23.4%, and 7.9% were categorized as underweight (BMI < 18.5), normal weight (BMI 18.5–24.9), overweight (BMI 25–29.9), and obese (BMI ≥ 30), respectively. For sample characteristics clustered for females and males, see Table 1.

Highest educational attainment is presented in Table 2 and occupation in Table 3.

### 3.2. Inferential Statistics

#### 3.2.1. Transport-related PA (TRPA)

##### Transport-Related Walking

Walking minutes per week, and the respective MET-minutes, decreased significantly demonstrating a small effect size (*p* < 0.001, d = 0.46), cf. Table 4.

##### Transport-Related Cycling

Cycling minutes per week, and the respective MET-minutes decreased significantly (*p* < 0.001, d = 0.30), cf. also Table 5.

TRPA (walking and cycling) also decreased significantly and showed moderate effect size (*p* < 0.001, d = 0.51), cf. Table 6.

#### 3.2.2. Leisure Time Activity (LTPA) and Muscle-Strengthening Activities (DMSA)

Time spent in LTPA decreased significantly (*p* < 0.001) with a small effect size (d = 0.31). The number of days of muscle-strengthening activities decreased slightly but not significantly on average by 0.1 days (*p* = 0.707), and with a trivial effect size (d = 0.01) (Table 7).

#### 3.2.3. Compliance with WHO PA Recommendations

Compliance with both parts of the WHO recommendations dropped significantly (aerobic activity from 49.7% to 31.1% (chi^2^ (1, 3000) = 107.789, *p* < 0.001, V = 0.19); and muscle-strengthening activity from 50.3% to 40.4% (chi^2^ (1, 3000) = 29.462, *p* < 0.001, V = 0.10) in lockdown conditions. The compliance with the combined recommendations decreased from 34.9% to 24.6% (chi^2^ (1, 3000) = 38.306, *p* < 0.001, V = 0.11).

**Figure 1:**
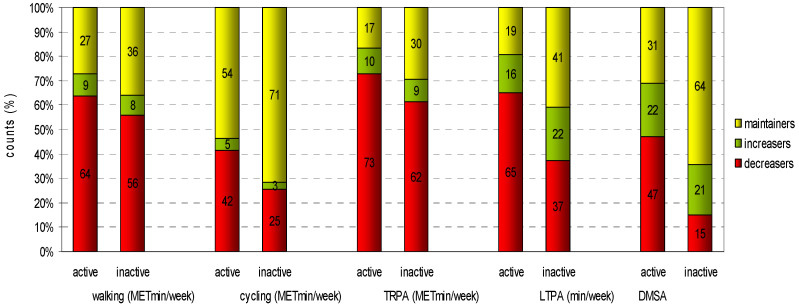
Counts (%) changes from normal to lockdown in the active and the inactive subsample.

Under normal conditions, 524 individuals fulfilled the combined WHO PA recommendation (active), and 976 individuals did not (inactive). The counts and percentage proportions (%) of changes (decreasers, increasers, maintainers) from normal to lockdown condition for the above-mentioned variables partly differ markedly between active and inactive respondents, with a lower rate of decreasers among the formerly inactive persons (Table 8 and Figure 1).

#### 3.2.4. Binary Logistic Regression Analysis

Binary logistic regressions were computed for the explanation of compliance with WHO PA recommendations using demographic data (sex: male vs. female, age, and BMI) as well as activity data (WRPA, TRPA, LTPA, and DMSA) and the categorical variable “lockdown vs. normal” condition. Due to missing demographic data (*n* = 11), 1489 datasets were analysed.

The regression model explained 70.1% of the total variance of compliance with the WHO recommendations (R^2^Nagelkerke = 0.730) with a correct estimation of 88.4%. The predictors sex (*p* = 0.006), LTPA, and DMSA (*p* < 0.001), as well as the “lockdown vs. normal” categorical variable (*p* = 0.002) showed a significant contribution, while age (*p* = 0.332), BMI (*p* = 0.067), TRPA (*p* = 0.605), and WRPA (*p* = 0.790) did not. Odds ratios were negligible for the non-significant parameters and LTPA, but relevant for DMSA and the lockdown condition (Table 9). For the categorical “lockdown vs. normal” variable (OR 0.640, 95% CI: 0.484–0.845) the odds ratio revealed a reduced chance (−36.0%) to comply with WHO PA recommendations under the lockdown condition (*p* = 0.002). For the categorical variable sex, the odds ratio revealed a reduced chance (−35.6%) for women to comply with WHO PA recommendations under the lockdown condition (*p* = 0.006). The variable DMSA demonstrated a highly increased chance (+117.1%) to comply with WHO PA recommendations.

## 4. Discussion

The aim of our study was to assess how the lockdown measures, which included the closure of PA infrastructure and the restrictions on leaving one’s home, affected PA at the population level in Italy during the first wave of the COVID-19 pandemic.

In our sample, all measures of PA showed a measurable decline during the lockdown period compared to the time prior to restrictions. We found the highest effect sizes in TRPA, in which domain PA basically halved (MET-minutes reduced by 52%). Almost two-thirds of respondents (65.5%) reported reduced TRPA in lockdown, which is easily explained by the closure of all non-essential businesses and the wide use of home offices. We also observed a marked (approx. 30%) decline in LTPA, which might be attributable to the closure of leisure time and sports facilities, such as fitness studios and swimming pools. Only the change in muscle-strengthening activities showed negligible changes.

Compliance with both aerobic and muscle-strengthening parts of the WHO recommendations decreased from about one-third of respondents to about one-fourth. We also observed that previously active and inactive participants changed their habitual PA differentially. About one in five previously inactive participants increased their LTPA and muscle-strengthening activities during lockdown. Only about one-half and one-third of previously active participants could increase or maintain their level of muscle-strengthening and LTPA, respectively. The regression analysis showed a higher chance for women than men for failing to comply with recommendations during lockdown. However, in our sample, age was not associated with an activity level according to the recommendations.

These data are, generally speaking, in line with other studies conducted in Italy and other countries. However, it has to be noted that direct comparison is limited by methodological differences, such as different study populations, instruments, and definitions of sufficient PA used and statistical analyses applied [24,25,26,27,28,29,30].

A survey among Italian university students using the International Physical Activity Questionnaire (IPAQ) [31] and an adapted version of the Adult Sedentary Behaviour Questionnaire (ASBQ) [32] found a significant overall reduction in PA and a significant increase in sedentary behavior, both with a large effect size during lockdown [28]. At the same time, 44.7% of respondents were able to maintain the minimum of 150 min of moderate intensity PA (IPAQ does not explicitly capture muscle-strengthening activities) [28]. Another study in the general population also using IPAQ found a significant decrease in overall, moderate, and vigorous PA, as well as walking during lockdown in a sample of 2524 participants of all ages, without reporting effect sizes [29]. The magnitude of the overall reduction was significantly larger in men than in women, but there were no differences among the four age groups [29]. Whereas participants classified as highly or moderately active prior to lockdown reduced their PA, low active participants increased it [29]. Di Renzo et al. examined the change in diet and lifestyle during the lockdown [27]. PA was assessed using four items of a previous survey, without any data on validity [27]. According to this study, 38.3% of respondents reported slightly increased PA [27]. A further study conducted in Northern Italy found that of the 490 adults (84% female), 50% of previously active respondents reduced their activity level and 27% of previously inactive respondents took up exercise [33]. Bourdas and Zacharakis found an overall 16.3% reduction in PA in a large sample (n = 8495) of Greeks, with the most pronounced reductions in previously highly active respondents [30]. Based on the multi-country survey, Wilke et al. also reported substantial reductions in PA levels, with the higher reductions in previously (highly) active participants [24].

The significance of reduced PA over a long period of time is clearly shown in so-called step reduction studies, which probably best mimic the current situation for large parts of the general population [17,34]. These intervention studies impose reduced ambulatory activities for typically one to two weeks. Reducing the number of steps taken from about 10,000 steps/day, approximating the recommended PA level, to a low level (less than 2500 steps/day) for 14 days induces detrimental metabolic adaptations, such as increased intra-abdominal and ectopic fat accumulation and hyperinsulinemia, even in young healthy adults [17,34]. Decline in ambulatory activity also leads to a loss of cardiorespiratory fitness, as much as 6.6% oxygen consumption mL/min/kg, as well as muscle atrophy in the legs [17]. In elderly participants, two weeks of step reduction impairs glucose and insulin metabolism, as well as skeletal muscle protein synthesis, and leads to muscle mass loss and increase in inflammatory cytokines [17,34]. Increasing step counts to pre-reduction levels can reverse the detrimental effects [17], and even low intensity resistant exercise in parallel with reduced ambulatory activity can contribute to the preservation of anabolic and insulin sensitivity [35]. However, this reversion might be partial or require longer and more intensive activity periods in the elderly and chronically ill than in younger healthy adults [17,34,36].

The relevance of PA goes far beyond metabolic health. The mental, psychological, and social benefits of PA in the general population under “normal circumstances” are unequivocal [37,38]. It has now been shown that PA can ease the psychological stress associated with the COVID-19 pandemic and can buffer the detrimental effects in different populations [39,40].

The results of the regression analysis underline the importance of muscle-strengthening activities. Muscle mass, strength, and quality are crucially important in cardiometabolic and musculoskeletal health throughout the lifespan [41,42]. As such, muscle-strengthening activities are now part of PA recommendations worldwide [16,43]. Nonetheless, (scientific) focus still tends to be on aerobic activities, which is also reflected in the fact that many studies on lockdown-related PA did not explicitly assess muscle-strengthening activities, and equated compliance or non-compliance with guidelines with performing or failing to perform only the required amount of aerobic activity [24,28]. It is plausible that without fitness studios, many people found it challenging to perform muscle-strengthening activities. Pragmatic forms of muscle-strengthening activities, such as using one’s own body weight or elastic bands, offer wide-ranging health benefits and can also be performed at home [44,45]. Individualized home-based exercise programs are also effective in improving balance, mobility, and muscle strength [46]. Another alternative way to perform muscle-strengthening activities is the use of outdoor fitness equipment in parks and other green areas. The regular use of such equipment can improve gait-related parameters, such as maximum isometric leg extensors force and gait velocity [47]. Accordingly, these approaches should be explicitly promoted by public health organizations.

Habitual PA volume has been shown to be affected by the seasons, such that it tends to be lower in winter and in periods with high levels of precipitation [48,49]. The first lockdown was during a spring period (March to May), which typically represents good weather conditions in Italy. It cannot be ruled out that, due to seasonal effects, current (autumn and winter) lockdowns might have an even more adverse influence on PA levels.

A strength of our study is the use of the validated EHIS questionnaire which, unlike IPAQ, for example, captures muscle-strengthening activities and thus reflects the current WHO PA recommendations more precisely, and which has been applied in all countries of the European Union. Nonetheless, our data are based on self-reporting and as such, are not free from reporting bias and the effects of social desirability. Furthermore, we assessed PA only once, which might have led to inaccurate reporting. Our sample size is reasonably large, but not representative, with far more women than men respondents. It cannot be ruled out that people with an interest in PA and health participated in the survey more readily.

## 5. Conclusions

In line with previous studies in Italy and in other countries, we report here a considerable decline in habitual PA in a large sample of Italians during the first COVID-19-related lockdown in spring 2020. If reductions are sustained or recurrent, detrimental somatic and mental health effects are probable. Since a complete return to normalcy will most likely not be for a considerable amount of time, it is essential that PA in general, and muscle-strengthening activities in particular, are promoted in the general population to limit the detrimental health effects of insufficient PA.

## Figures and Tables

**Table 1:** Sample characteristics.

Characteristics		*n*	Mean ± SD	Range
Age (yr.)	females	1124	42.7 ± 10.6	15–67
	males	367	44.2 ± 12.9	15–76
Weight (kg)	females	1121	63.2 ± 11.5	38–123
	males	368	79.6 ± 13.0	50–145
Height (m)	females	1124	1.65 ± 0.06	1.47–1.88
	males	368	1.77 ± 0.07	1.57–1.99
BMI (kg/m^2^)	females	1121	23.1 ± 4.0	14.5–45.7
	males	368	25.3 ± 3.8	17.4–46.3

BMI = body mass index.

**Table 2:** Highest educational attainment.

Highest Educational Attainment	*n* (%)
Missing	8 (0.5)
Primary education	8 (0.5)
Lower secondary education	154 (10.3)
Higher secondary education	589 (39.3)
Post-secondary non-tertiary education	25 (1.7)
Short-cycle tertiary education	32 (2.1)
Bachelor’s or equivalent level	196 (13.1)
Master’s or equivalent level	447 (29.8)
Doctoral or equivalent level	41 (2.7)
Total	1500 (100)

**Table 3:** Occupation.

Occupation	*n* (%)
Missing	24 (1.6)
Armed forces	17 (1.1)
Managers	37 (2.5)
Professionals	448 (29.9)
Technicians and associate professionals	63 (4.2)
Clerical support workers	394 (26.3)
Service and sales workers	164 (10.9)
Skilled agricultural, forestry, and fishery workers	5 (0.3)
Craft and related trades workers	66 (4.4)
Plant and machine operators and assemblers	6(0.4)
Elementary occupations	59 (3.9)
Pensioners	12 (0.8)
Students	135 (9.0)
Unemployed	70 (4.7)
Total	1500 (100)

**Table 4:** Walking minutes per week and walking MET-minutes per week.

Walking		N	Mean	SD	SEM	Mean Diff	t-Value	*p*-Value	Cohen’s D	95% CI Lower	95% CI Upper
Walking (min/week)	normal	1500	216.2	271.8	7.0	104.4	17.882	<0.001	0.46	92.9	115.8
	lockdown	1500	111.8	197.7	5.1						
Walking (METmin/week)	normal	1500	713.4	896.8	23.2	344.4	17.882	<0.001	0.46	306.6	382.2
	lockdown	1500	369.0	652.4	16.8						

METmin/week = metabolic equivalent of task minutes per week.

**Table 5:** Cycling minutes per week and cycling MET-minutes per week.

Cycling		*N*	Mean	SD	SEM	Mean Diff	t-Value	*p*-Value	Cohen’s D	95% CI Lower	95% CI Upper
Cycling (min/week)	normal	1500	39.3	96.7	2.5	24.754	11.675	<0.001	0.30	20.6	28.9
	lockdown	1500	14.6	62.0	1.6						
Cycling (METmin/week)	normal	1500	235.9	580.1	15.0	148.5	11.675	<0.001	0.30	123.6	173.5
	lockdown	1500	87.4	372.2	9.6						

METmin/week = metabolic equivalent of task minutes per week.

**Table 6:** Transport-related activity MET-minutes per week.

Transport-Related Activity		N	Mean	SD	SEM	Mean Diff	t-Value	*p*-Value	Cohen’s D	95% CI Lower	95% CI Upper
TRPA (METmin/week)	normal	1500	949.3	1158.0	29.9	492.9	19.705	<0.001	0.51	443.9	542.0
	lockdown	1500	456.3	819.1	21.1						

TRPA = transport-related physical activity, METmin/week = metabolic equivalent of task minutes per week.

**Table 7:** Leisure time activity.

Leisure time activity		*N*	Mean	SD	SEM	Mean Diff	t-Value	*p*-Value	Cohen’s D	95% CI Lower	95% CI Upper
LTPA (min/week)	normal	1500	160.8	175.7	4.5	48.2	12.147	<0.001	0.31	40.4	56.0
	lockdown	1500	112.6	150.4	3.9						
DMSA	normal	1500	1.76	1.90	0.05	0.02	0.367	0.707	0.30	−0.08	0.11
	lockdown	1500	1.74	2.21	0.06						

LTPA = leisure time physical activity, DMSA = days of muscle-strengthening activities per week.

**Table 8:** Counts (%) changes from normal to lockdown in the total, the active and the inactive subsample.

Activity		Total (*n* = 1500)		Active (*n* = 524)		Inactive (*n* = 976)	
		Counts	(%)	Counts	(%)	Counts	(%)
walking (METmin/week)	decreasers	878	58.5	334	63.7	544	55.7
	increasers	129	8.6	48	9.2	81	8.3
	maintainers	493	32.9	142	27.1	351	36.0
cycling (METmin/week)	decreasers	466	31.1	218	41.6	248	25.4
	increasers	56	3.7	25	4.8	31	3.2
	maintainers	978	65.2	281	53.6	697	71.4
TRPA (METmin/week)	decreasers	983	65.5	382	72.9	601	61.6
	increasers	141	9.4	54	10.3	87	8.9
	maintainers	376	25.1	88	16.8	288	29.5
LTPA (min/week)	decreasers	704	46.9	341	65.1	363	37.2
	increasers	297	19.8	82	15.6	215	22.0
	maintainers	499	33.3	101	19.3	398	40.8
DMSA	decreasers	393	26.2	247	47.1	146	15.0
	increasers	316	21.1	114	21.8	202	20.7
	maintainers	791	52.7	163	31.1	628	64.3

TRPA = transport-related physical activity, LTPA = leisure time physical activity, DMSA = days of muscle-strengthening activities per week, METmin/week = metabolic equivalent of task minutes per week.

**Table 9:** Binary logistic regression model with odds ratios (95% CI) for the explanation of compliance with WHO PA recommendations including sex, age, BMI, WRPA, TRPA, LTPA, and DMSA, and the categorical variable “lockdown vs. normal” condition.

		B	SE	Wald	DF	Sig.	Exp(B)	95% CI EXP(B)	
								Lower	Upper
Step 1	Lockdown (lock = 0/norm = 1)	−0.447	0.142	9.901	1	0.002	0.640	0.484	0.845
	Sex	−0.440	0.162	7.436	1	0.006	0.644	0.469	0.884
	Age	0.006	0.006	0.940	1	0.332	1.006	0.994	1.017
	BMI	−0.035	0.019	3.347	1	0.067	0.966	0.930	1.003
	WRPA	−0.029	0.109	0.071	1	0.790	0.971	0.784	1.203
	TRPA	0.000	0.000	0.267	1	0.605	1.000	1.000	1.000
	LTPA	0.016	0.001	455.167	1	0.000	1.016	1.015	1.018
	DMSA	0.775	0.041	359.145	1	0.000	2.171	2.004	2.352
	constant	−4.278	0.511	70.006	1	0.000	0.014		

BMI = body mass index, LTPA = leisure time physical activity, TRPA = transport-related physical activity, DMSA = days of muscle-strengthening activities per week, WRPA = work-related physical activity.

## Data Availability

The data presented in this study are available on request from JS.
